# Validation and Uncertainty Estimation of an Ecofriendly and Stability-Indicating HPLC Method for Determination of Diltiazem in Pharmaceutical Preparations

**DOI:** 10.1155/2013/353814

**Published:** 2013-09-17

**Authors:** Fahimeh Sadeghi, Latifeh Navidpour, Sima Bayat, Minoo Afshar

**Affiliations:** ^1^Department of Pharmaceutics, Pharmaceutical Sciences Branch, Islamic Azad University (IAUPS), Tehran 193956466, Iran; ^2^Department of Medicinal Chemistry, Faculty of Pharmacy, Tehran University of Medical Sciences, Tehran 14176, Iran

## Abstract

A green, simple, and stability-indicating RP-HPLC method was developed for the determination of diltiazem in topical preparations. The separation was based on a C_18_ analytical column using a mobile phase consisted of ethanol: phosphoric acid solution (pH = 2.5) (35 : 65, v/v). Column temperature was set at 50°C and quantitation was achieved with UV detection at 240 nm. In forced degradation studies, the drug was subjected to oxidation, hydrolysis, photolysis, and heat. The method was validated for specificity, selectivity, linearity, precision, accuracy, and robustness. The applied procedure was found to be linear in diltiazem concentration range of 0.5–50 **μ**g/mL (*r* ^2^ = 0.9996). Precision was evaluated by replicate analysis in which % relative standard deviation (RSD) values for areas were found below 2.0. The recoveries obtained (99.25%–101.66%) ensured the accuracy of the developed method. The degradation products as well as the pharmaceutical excipients were well resolved from the pure drug. The expanded uncertainty (5.63%) of the method was also estimated from method validation data. Accordingly, the proposed validated and sustainable procedure was proved to be suitable for routine analyzing and stability studies of diltiazem in pharmaceutical preparations.

## 1. Introduction

**Figure 1:**
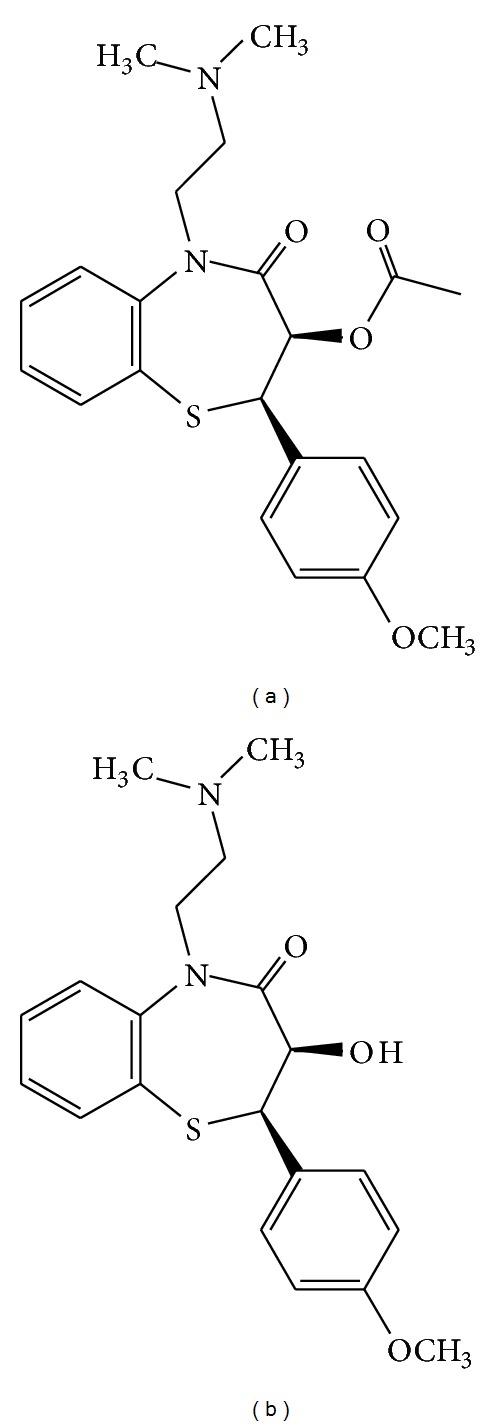
Molecular structures of diltiazem (a) and desacetyl diltiazem (b).

Anal fissure is one of the most common and painful proctologic diseases that is effectively treated and prevented with conservative measures in its acute form, whereas chronic fissures may require medical or surgical therapy. Because of the disability associated with surgery for anal fissure and the risk of incontinence, medical alternatives for surgery have been sought. Most recently, pharmacologic approaches that relax the anal smooth muscle, to accomplish reversibly what occurs in surgery, have been used to obtain fissure healing [1–3]. Glyceryl trinitrate ointment (0.2%) has an efficacy of up to 68% in healing chronic anal fissure, but it is associated with headache as the major and most common side effect. Diltiazem, a calcium channel antagonist used in the treatment of angina, hypertension, and arrhythmias, achieved healing of chronic anal fissure comparable to 0.2% Glyceryl trinitrate ointment but caused fewer side effects [4]. Therefore, it is preferred to use diltiazem to treat this chronic disease [5]. The structures of diltiazem and its main impurity are presented in Figure 1.

Concerning new therapeutic application of diltiazem, validated and stability-indicating methods should be available to determine this drug in topical preparations. The United States and British Pharmacopeias present two reversed-phase HPLC methods using d-10-comphorsulfonic acid and *N,N*-dimethyloctylamine, respectively, as amine modifiers suitable for the quantitative analysis of diltiazem in raw material and tablet formulation. However, the use of such ionic additives has fallen somewhat out of favor, as these can have some undesirable effects, including the difficulty of their removal from the stationary phase after use, leading to permanent alteration of its properties and even chemical reaction with some solute types [6]. Moreover, no method was compiled in these pharmacopoeias for the analysis of this medicine in topical preparations. In addition, literature survey reveals little information about the quantitative determination of diltiazem in topical formulations and there is only one report dealing with the analysis of diltiazem in a transdermal gel which has not been validated according to International Conference on Harmonization (ICH) stability-indicating guidelines [7].

Furthermore, in all of the previously reported HPLC methods for determination of diltiazem in pharmaceutical or biological matrixes with UV detection, acetonitrile or methanol has been used as a part of mobile phase or extraction procedure [7–13]. It is worth mentioning that these solvents are ranked by US Environmental Protection Agency (EPA) as hazardous solvents [14] and because of their inherent toxicity [15], safe detoxification of the waste solvents is essential, which may lead to high to very high disposal costs. In recent years, green analytical chemistry has recognized momentum not only in the academic world but also in industrial and pharmaceutical laboratories. The literature of green chemistry has undergone a drastic increase. Green analytical techniques aim to minimize or eliminate the hazardous waste associated with analytical methods [16–20]. Moreover, from an economic point of view, it is reasonable to replace acetonitrile by other solvents (preferably ecofriendly ones) because of its worldwide shortage [21–24].

Testing laboratorie, who wish to comply with the requirements of ISO/IEC 17025:2005 need to estimate uncertainty of measurement for their quantitative methods. Estimation of uncertainty leads to better measurement reliability, renders data from interlaboratory studies comparable, and helps to assess the statistical significance of the difference between the measurement and a relevant reference value [25, 26].

Although few methods for the determination of diltiazem in pharmaceutical matrixes have been reported, information on such metrological parameters is scarce [7–9, 11].

Taking ICH guidelines into consideration [27], the present study describes a simple, validated, and stability-indicating analytical method for the determination of diltiazem in pharmaceutical preparations, which meets the green aspects in analytical chemistry. Also, the calculation of the measurement uncertainty which is based on the validation of the analytical procedures in a laboratory is presented. Moreover, the performance of the method was evaluated and its potential for the determination of diltiazem in pharmaceutical preparations was investigated.

## 2. Experimental Section

### 2.1. Materials, Reagents, and Chemicals

Qualified diltiazem hydrochloride standard (99.50%) and pharmaceutical grade diltiazem hydrochloride were kindly provided by Arya pharmaceuticals (Tehran, Iran). All solvents and reagents were of gradient and analytical grade, respectively, and were purchased from Merck (Darmstadt, Germany). Desacetyl diltiazem was synthesized according to the previously reported method [28]. Identification test of desacetyl diltiazem was performed with IR and NMR spectrometry (data not shown). HPLC-grade water was obtained through a Milli-Q system (Millipore, Milford, MA, USA) and was used to prepare all solutions. The pharmaceutical formulations (gel 2%) and the corresponding placebos (mixture of all the excipients) were prepared in our laboratory. Diltiazem organogel 2% manufactured by Troikaa Pharmaceuticals Ltd. (India) was purchased from a local pharmacy.

#### 2.1.1. Preparation of Standard Solutions

Stock standard solution of diltiazem was prepared in methanol at a concentration of 1 mg/mL. This solution was found to be stable for at least 1 month, when it was stored at 2–8°C. Freshly prepared working standards at concentration levels of 0.5, 1, 5, 10, 20, and 50 *μ*g/mL were obtained from stock solution by the appropriate dilution in HPLC-grade water.

#### 2.1.2. Preparation of Test Solutions

A 1.0–1.5 g portion of gel (equivalent to 20–30 mg of diltiazem) was transferred into a 25 mL volumetric flask. Then, 15 mL of HPLC-grade water was added to this portion and the solution was sonicated for 10 min. Thereafter the volume was adjusted to the mark with the same medium to provide a theoretical concentration of 800–1200 *μ*g/mL of diltiazem. This solution was again diluted with HPLC-grade water to make final concentration of 8–12 *μ*g/mL. The experiment was performed in triplicate. These samples were assayed using calibration curves of working standard solutions. The same procedure was applied to placebo to be sure about the selectivity of the method.

### 2.2. HPLC Analysis

The HPLC method was carried out on a Shimadzu HPLC system (Shimadzu, Kyoto, Japan), set to recycle the mobile phase and was equipped with an SCL-10AVP system controller, LC-10 ADVP pump, DGU-14A degasser, and a SPD-M10AVP PDA detector. The peak areas were integrated automatically by computer using a Shimadzu Class VP software program. A 20 *μ*L volume of sample was introduced into a Rheodyne model 7725i injector.

The elution was carried out on a C_18_ column (250 mm × 4.6 mm, 5 *μ*m particle size) from Hector (Daejeon, South Korea). All analyses were performed at the column temperature of 50 ± 1°C under isocratic conditions with a mobile phase of ethanol : phosphoric acid solution (pH = 2.5) (35 : 65, v/v) and a flow rate of 2.0 mL/min, using PDA detection at 240 nm.

### 2.3. Forced Degradation Studies

The stability-indicating capability of the method was determined by subjecting diltiazem solutions (standard and pharmaceutical preparations) at the concentration level of 100 *μ*g/mL to accelerated degradation by acidic, basic, heat, oxidative, and photolytic conditions to evaluate the interferences in the quantitation of diltiazem. Sample solutions prepared in 1 M hydrochloric acid and 1 M sodium hydroxide were used for the acidic and basic hydrolysis, respectively. Both solutions were heated at 70°C for 12 h and then neutralized with basic or acidic solutions, as necessary. For evaluating the heat condition, the sample solutions were heated at 70°C for 12 h. For oxidative degradation, sample solutions were exposed to a solution of hydrogen peroxide (3%) and kept at ambient temperature for 4 h, protected from light. Photodegradation was induced by exposing the sample solution to UV-Lamp at a wavelength of 254 nm placed in a wooden cabinet for 4 hours. The experiments were performed in triplicate. The solutions were diluted with HPLC-grade water to final concentration of 10 *μ*g/mL and were injected into chromatograph.

### 2.4. Method Validation

The developed method was validated as per the requirements of the ICH guidelines [27]. Linearity was evaluated by determining six working standard solutions at a concentration range of 0.5–50 *μ*g/mL. Five sets of such solutions were prepared. Each set was analyzed to plot a calibration curve. Slope, intercept, and coefficient of determination (*r* ^2^) of the calibration curves were calculated to ascertain linearity of the method. The limit of quantification (LOQ) was defined as the lowest concentrations with the RSDs lower than 5% and accuracies within ±5%, considering at least ten times the response compared to that of the blank. In order to check the robustness, the effect of small but deliberate variations in the chromatographic conditions was evaluated. The conditions studied were flow rate (altered by ±0.2 mL/min), column temperature (altered by ±2°C), and pH of phosphoric acid solution (altered by ±0.2). These chromatographic variations were evaluated for resolution between diltiazem and desacetyl diltiazem, % assay of the drug, theoretical plates, and tailing factors of the peaks. For method repeatability, assay of working standard solutions (0.5, 5, 10, and 50 *μ*g/mL) was repeatedly performed five times on the same day (intraday). For reproducibility, freshly prepared solutions at aforementioned concentration levels were analyzed at different days (interday) and results were statistically evaluated in terms of % RSD. For recovery studies, 0.125 g portions of preassayed diltiazem gel 2% were spiked with extra 0.5, 1, and 2 mL of stock standard solution. These samples were handled as explained in Section 2.1.2 and the final target levels of 12, 14, and 18 *μ*g/mL were prepared. The concentrations were calculated using calibration curves. Accuracy was calculated as the deviation of the mean from nominal concentration. To assess accuracy, freshly prepared placebo of the diltiazem pharmaceutical formulations was spiked with various amounts of diltiazem to obtain the concentration levels of 0.5, 5, 10, and 50 *μ*g/mL. Each solution was injected in triplicate. Selectivity of this method was indicated by the absence of any excipient interference at retention times of the peaks of diltiazem. The absence of interfering peak was evaluated by injecting a blank sample consisting of diluent and placebo. The double check of the lack of interferences of the resulting by-products with the elution of the peaks of diltiazem was done by calculating the *F* factor, meaning the ratio of the UV molar absorption coefficients of diltiazem at the 240 (peak) and 260 (valley) nm, respectively, using

(1) $$\begin{array}{c} F=\frac{A\left(240\right)}{A\left(260\right)}, \end{array}$$

where *A*(240) and *A*(260) are the diltiazem peaks areas obtained at 240 and 260 nm, respectively. The resulting *F* factors were compared with that of the standard [29]. Moreover, the UV spectrum of each diltiazem peak was acquired during the appearance of the peak in the chromatogram.

### 2.5. Estimation of the Uncertainty of the Measurements

An expanded uncertainty budget was constructed for diltiazem in pharmaceutical preparations by the RP-HPLC-PDA method according to previously reported procedures [26, 30–32].

Four individual sources were taken into account to assess the expanded uncertainty.

#### 2.5.1. Uncertainty of the Measurement Standard

The uncertainty of the measurement standard is calculated by the quadratic addition of two terms: the uncertainty certified by manufacturer (*U*_stock_) and the uncertainty corresponding to its preparation by dilution or weighting (*U*_preparation_) [30]:

(2) $$\begin{array}{c} U_{\text{standard}}\left(\%\right)=\sqrt{U_{\text{stock}}^{2}\left(\%\right)+U_{\text{preparation}}^{2}\left(\%\right)}. \end{array}$$

The stock uncertainty (*U*_stock_) is calculated from a value given by the manufacturer using

(3) $$\begin{array}{c} U_{\text{stock}}\left(\%\right)=\frac{\left(100-P\%\right)}{\sqrt{3}}, \end{array}$$

where the purity is expressed as *P*%. When there are independent standard preparations at each concentration level, the *U*_preparation_ term could be eliminated. In this case, the contribution of this term is included in the *U*_preparation_ term [26, 30].

#### 2.5.2. Uncertainty Associated to the Calibration Curve

This value is calculated using [31]

(4) $$\begin{array}{c} U_{\text{calibration}}=\frac{s_{x_{0}}}{x_{0}}, \end{array}$$

where *x*_0_ is the concentration calculated from the calibration curve and *s*_*x*_0__ is the standard deviation of the concentration, obtained from the calibration curve using

(5) $$\begin{array}{c} s_{x_{0}}=\frac{s\left(r\right)}{m}\sqrt{\frac{1}{N}+\frac{1}{n}+\frac{{\left({\overset{-}{\Upsilon }}_{0}-\overset{-}{\Upsilon }\right)}^{2}}{m^{2}\sum_{i=1}^{n}{\left(x_{i}-\overset{-}{x}\right)}^{2}},}\\ s\left(r\right)=\sqrt{\frac{\sum {\left(y_{i}-mx_{i}-b\right)}^{2}}{n-2}}, \end{array}$$

where *m* is the slope of the line, *b* is the *y*-intercept of the line, *N* is the number of replicate unknowns, *n* is the number of the standards, ${\overset{-}{\Upsilon }}_{0}$ is the mean of *N* repeat measurements of *y* for the sample, $\overset{-}{\Upsilon }$ is the mean of the *y* values for the calibration standards, *x*_*i*_ are the concentrations of the standards, and $\overset{-}{x}$ is the average concentration of the standards.

#### 2.5.3. Uncertainty Associated to Precision

This value is calculated using

(6) $$\begin{array}{c} U_{\text{precision}}=\frac{s}{x_{0\sqrt{n}}}, \end{array}$$

where *s* is the standard deviation of the experimental data for precision and *n* is the number of assays [31].

#### 2.5.4. Uncertainty Associated to Accuracy

This parameter is calculated using (7):

(7) $$\begin{array}{c} U_{\text{accuracy}}=\frac{s\left(\eta \right)}{\sqrt{n}}, \end{array}$$

where *s*(*η*) is the relative standard deviation of the recovery and *n* is the number of assays [31].

The value of the expanded uncertainty was calculated according to ISO GUM guidelines using (8):

(8) $$\begin{array}{c} U=kc\sqrt{U_{\text{standard}}^{2}+U_{\text{calibration}}^{2}+U_{\text{precision}}^{2}+U_{\text{accuracy}}^{2}\;}, \end{array}$$

where *U* is expanded uncertainty, *k* the coverage factor (for confidence interval 95%, *k* = 2), and *c* is the concentration of the drug [32].

## 3. Results and Discussion

### 3.1. Optimization of the Chromatographic Conditions

The HPLC procedure was optimized with a view to develop a green stability-indicating assay method while keeping the system suitability necessities according to the United States Pharmacopeia, which needs the resolution between diltiazem and desacetyl diltiazem and the number of theoretical plates for the diltiazem peak to be greater than 3 and 1200, respectively.

**Figure 2:**
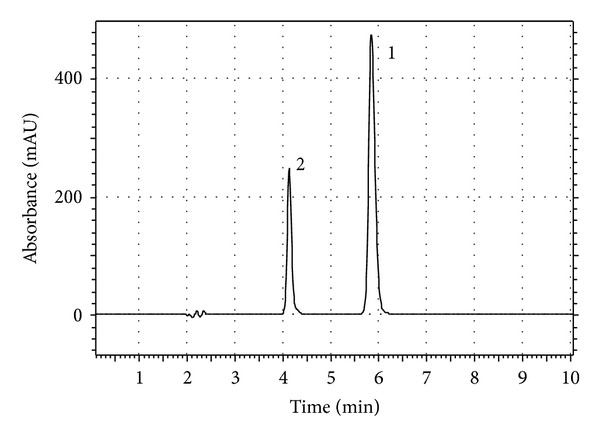
Typical chromatogram of diltiazem and its main impurity (desacetyl diltiazem); peak 2: desacetyl diltiazem, peak 1: diltiazem.

In order to follow the first principle of green chemistry, acetonitrile was replaced by ethanol in the previously reported mobile phase containing acetonitrile : sodium phosphate monobasic monohydrate buffer (pH 2.5, 0.02 M) (33 : 67 v/v), which was used for the analysis of diltiazem in a transdermal gel [7]. Although ethanol has some shared or similar characteristics to methanol and acetonitrile, including complete miscibility with H_2_O, availability in the high purity required for HPLC and low chemical reactivity with most sample species as well as with HPLC, instrument and column surfaces, it has some characteristics that are less favorable. One of them is that the viscosities of ethanol : H_2_O solutions are higher than those of methanol : H_2_O and acetonitrile : H_2_O solutions of the same elution strength at room temperature [33]. To cope with this problem the temperature was raised up to 50°C. This change resulted in lower chromatographic pressure, better peak shapes, good separation of diltiazem and desacetyl diltiazem, and suitable theoretical plates. However, the retention times were not satisfactory when the flow rate was 1 mL/min. Therefore, this parameter was increased to 2 mL/min to resolve the aforementioned problem. According to these preliminary results, the detection wavelength of 240 nm, flow rate of 2 mL/min and the mobile phase of phosphoric acid solution (pH = 2.5) : ethanol (65 : 35, v/v), and the column temperature of 50°C were selected. Under the chromatographic conditions of this method, desacetyl diltiazem and diltiazem were separated completely from each other and their peaks appeared at 4.2 and 5.8 min, respectively (Figure 2), the theoretical plates for diltiazem peak was 11511.26 ± 0.33%, the resolution between diltiazem and its impurity was more than 9, and the tailing factor for diltiazem peak was 1.1 ± 1.30%. The values of theoretical plates obtained in this study are much higher than those reported previously (1863 ± 4.65) [9] which indicates better column efficacies at the chromatographic conditions used in this study. Before fully implemented in the quantitative determination of drug substance and pharmaceutical preparation, this method was thoroughly validated according to ICH guidelines [27].

### 3.2. Forced Degradation Studies

**Figure 3:**
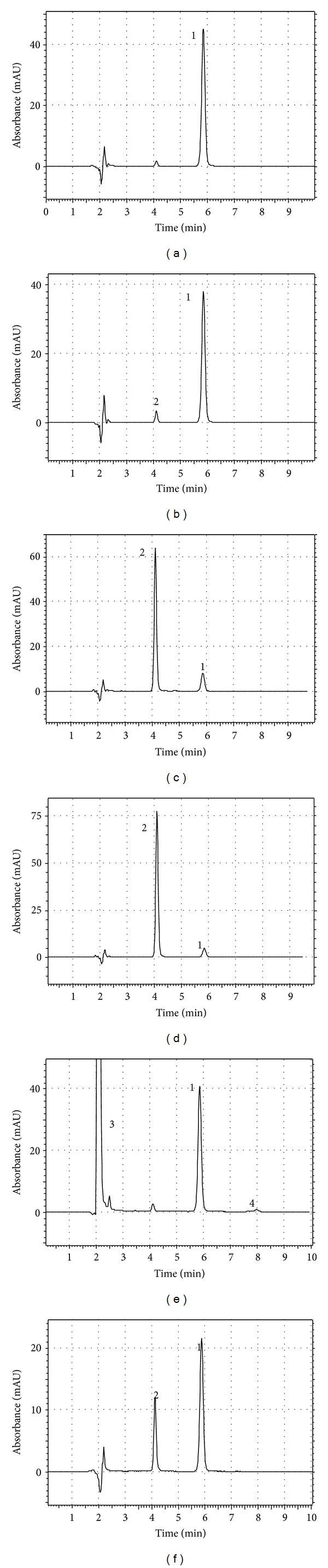
Typical chromatograms of (a) diltiazem working standard solution (10 *μ*g/mL) and after degradation under (b) heat condition; (c) acidic hydrolysis; (d) basic hydrolysis; (e) oxidative condition: peak 3: hydrogen peroxide, peak 4: oxidative impurity; (f) photolytic condition: peak 1: diltiazem, peak 2: desacetyldiltiazem.

Stability-indicating method is defined as an analytical method that accurately quantifies the active ingredients without interference from degradation products, process impurities, excipients, or other potential impurities [34]. Diltiazem showed drastic degradation in acidic, basic, and photolytic conditions; in that only 16.67 ± 1.95, 10.47 ± 2.10, and 48.86 ± 1.48% of the drug remained, respectively, and at the same time a remarkable increase in the concentrations of desacetyl diltiazem was observed (Figures 3(c), 3(d), and 3(f)). The first-order hydrolysis of diltiazem in the presence of acids and bases and formation of desacetyl diltiazen in these conditions has been reported previously, which confirms the results of this study [35]. Andrisano et al. showed that the main photoproduct of diltiazen after irradiation of UV-A and UV-B is diltiazem-*S*-oxide [36]. However, according to the findings of the present study the main photoproduct after exposure of an aqueous solution of diltiazem to UV-C is desacetyl diltiazem, indicating a different photodegradation process. Under the oxidative and heat conditions, the diltiazem contents decreased to 89.01 ± 1.74 and 84.57 ± 0.86%, respectively, with a minor increase in the amounts of desacetyl diltiazem (Figures 3(b) and 3(e)). The degradation products of the parent compound were found to be similar for both the pharmaceutical and standard solutions. All the degradation studies are summarized in Table 1.

### 3.3. Method Validation

#### 3.3.1. Selectivity and Specificity

**Figure 4:**
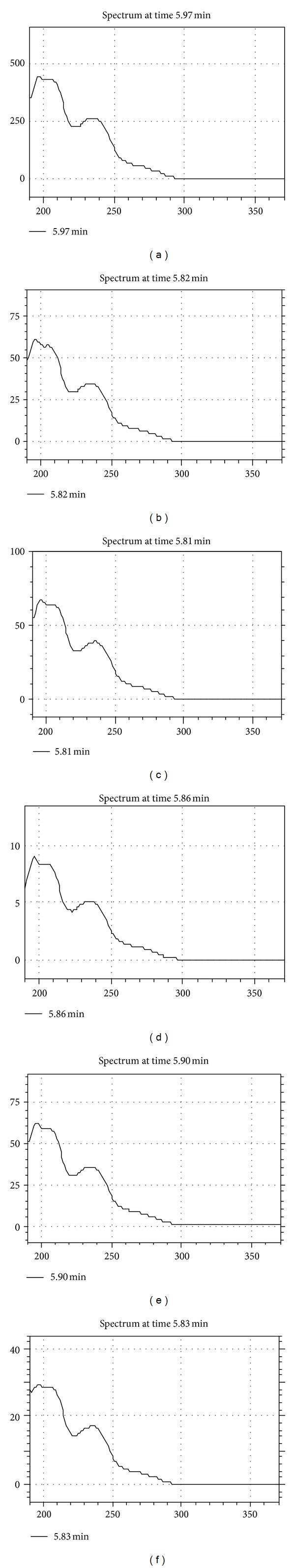
Typical spectrums of (a) diltiazem working standard solution and after degradation under (b) heat condition; (c) acidic hydrolysis; (d) basic hydrolysis; (e) oxidative condition; (f) photolytic condition.

Specificity is the ability of the method to unequivocally assess the analyte response in the presence of its potential impurities that was illustrated by the complete separation of diltiazem from degradation products as shown in Figure 3. Furthermore, the decreases observed in diltiazem contents in stability studies, when degradation products appeared, proved the specificity of the method (Table 1). Also, the *F* factors of the diltiazem in stress solutions (3.52–3.55) show the same values of that of the standard (3.55). The UV spectrum obtained during the appearance of the peaks also confirms their purity (Figure 4). Consequently, the forced degradation studies documented the stability-indicating power and specificity of the proposed method.

**Figure 5:**
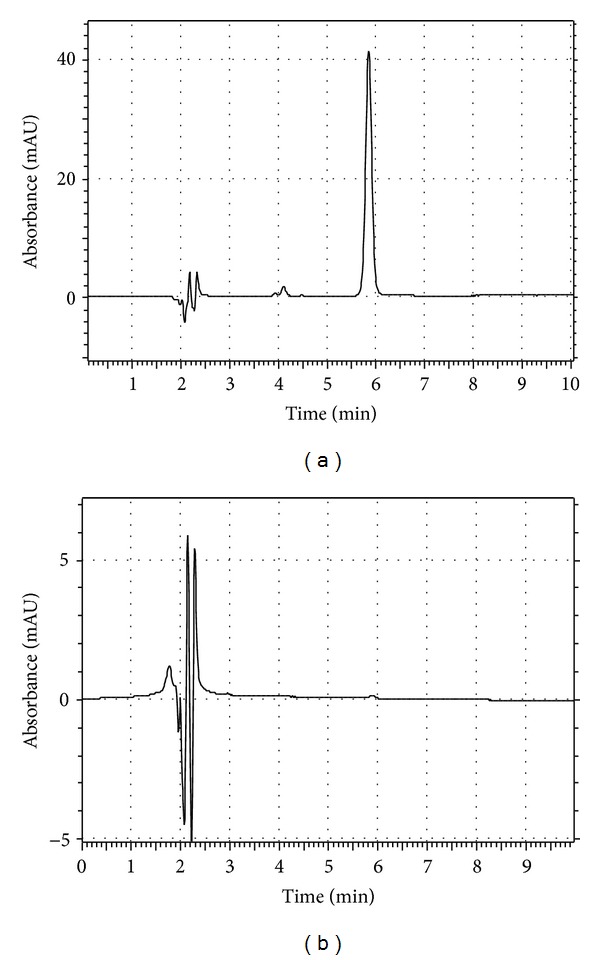
A chromatogram obtained from analyzing the commercially available gel. The solution contains the target diltiazem concentration of 9.35 *μ*g/mL. (b) blank.

The application of the whole procedure to placebo samples in order to verify the method selectivity demonstrates that no interferences were detected (Figure 5(b)).

#### 3.3.2. Linearity, Precision, and LOQ

Linearity was determined by constructing five independent calibration curves, each one with six calibration points of diltiazem, including the LOQ, in the range of 0.5–50 *μ*g/mL. The peak areas of diltiazem against the respective concentrations were used for plotting the graph, and the linearity was evaluated by the least square regression analysis. The linearity curve was defined by the following equation: *y* = 44446.17*x* + 5597.38 (*r* ^2^ = 0.9996), which indicated the linearity of the calibration curve for the method. Moreover, the relative standard error of slope can be used as a parameter with respect to the precision of the regression, as a general acceptance criterion for the linearity performance of the analytical procedure [37]. This parameter should be comparable to the calculated RSD in the evaluation of the precision. In this study, the result obtained for the RSD of the slopes was 1.22% which is comparable to mean value 1.36% of the RSD of the precision.

Summary of the method validation results is shown in Table 2. The method was proved to be precise, as the intra- and interday precision calculated for the concentration levels of 5, 10 and 50 *μ*g/mL ranged from 0.78% to 1.46% and from 1.18% to 1.53%, respectively. These values fulfill the validation criteria of an analytical method designed for quality control of pharmaceutical preparations for which RSD values <2% are acceptable [37].

The LOQ is the lowest concentration that can be quantified with acceptable precision and accuracy. The LOQ of diltiazem was determined to be 0.5 *μ*g/mL, considering the mean accuracy value of 96.67% and RSD value of 0.91% (Table 2). These values indicate that the proposed method is much more sensitive than what have been reported previously for analysis of diltiazem in a transdermal gel [7]. Diltiazem is a basic compound and consequently prone to peak tailing and poor peak efficiency; therefore, sensitivity of the method is of paramount importance to resolve the mentioned problem because one of the factors which can end up to peak asymmetry is sample overload [6].

#### 3.3.3. Recovery and Accuracy

The accuracy was evaluated applying the proposed method to the analysis of the in-house mixture of the gel excipients with known amounts of the drug, to obtain solutions at concentration levels of 0.5, 5, and 10 and 50 *μ*g/mL. The accuracy was assessed from three replicate determinations and calculated as the percentage of the drug recovered from the formulation matrix. The mean and RSD values calculated for the analysis of three diltiazem concentration levels of 5, 10, and 50 *μ*g/mL are shown in Table 2; the mean values were found to be 100.1%, 99.06%, and 98.78% with RSDs 1.32%, 0.67%, and 0.59%, respectively, demonstrating that the method is accurate within the desired range. Also, the results obtained from the analysis of preassayed pharmaceutical preparations, spiked with different amounts of diltiazem stock solution, revealed acceptable recoveries with the mean value of 100.49 and %RSDs < 1.61, respectively. These values document a high recovery in this method.

#### 3.3.4. Robustness

Chromatographic parameters including % of assay, resolution between diltiazem and its impurity, theoretical plates, and tailing factor of diltiazem peaks were not significantly affected by the slight changes in the chromatographic conditions like alteration in flow rates, pH of the aqueous solution of mobile phase, and column temperature. Analysis was carried out in triplicate and only one parameter was changed in the experiments at a time. The results of the experimental variables evaluated were within the acceptable deviation (RSD < 2%), the resolution of the aforementioned peaks was more than 9, and the theoretical plates and tailing factor parameters were calculated to be more than 11500 and less than 1.2, respectively, indicating that the proposed method is robust under the conditions tested.

#### 3.3.5. The Uncertainty of the Method

The expanded uncertainty of the method for quantification of diltiazem in pharmaceutical preparations was calculated. The results are shown in Table 3. Among the four sources of uncertainty, which were taken into consideration, the uncertainty associated with calibration appears to be the most important source in the overall uncertainty. Therefore, analysts should pay great attention when conducting calibrations of instrumental techniques.

#### 3.3.6. Application of the Method

The optimized and validated method was applied to the determination of diltiazem in marketed gels. The amount of diltiazem in gels was calculated using calibration curve method. Typical chromatogram obtained following the assay of the pharmaceutical dosage form is shown in (Figure 5(a)). The results of the assay undertaken and the calculated uncertainties are shown in Table 3. The value of 99.46% of label claim indicates that the method is selective for the analysis of diltiazem without interference from the excipients used to formulate and produce these gels.

## 4. Conclusion

The aim of this study was to develop a green and specific method for the determination of diltiazem in pharmaceutical preparations. The method was designed to be specific, selective, sensitive, robust, reproducible, accurate, inexpensive, and easy to perform. The principal advantage of the method is the use of available environmentally friendly solvents and reagents for LC analyzing and extractions to follow the first principle of green chemistry which emphasizes waste prevention instead of remediation [15]. To the best of our knowledge, this is the first method which is thoroughly green and reports the metrological parameters in quantification of diltiazem in pharmaceutical dosage forms. In addition, recycling significantly reduced the mobile phase consumption and made the method economic. Moreover, the method is much more sensitive than the previously reported procedure [7].

Finally, the newly developed method was successfully performed for the analysis of diltiazem in pharmaceutical preparations and it can thus be used for routine analysis, quality control, and studies of the stability of pharmaceutical formulations containing diltiazem.

## Figures and Tables

**Table 1:** Summary of stress degradation studies of diltiazem.

Stress condition/media/duration	Recovered diltiazem (%) mean (RSD, %)	No. of observed impurities
Photolytic/H_2_O/254 nm/4 h	48.86 (1.48)	1
Acidic/1.0 N HCl/70°C/12 h	16.67 (1.95)	1
Neutral/H_2_O/70°C/12 h	84.57 (0.86)	1
Oxidative/3.0% H_2_O_2_/4 h	89.01 (1.74)	1
Basic/1.0 N NaOH/70°C/12 h	10.47 (2.10)	2

**Table 2:** Precision, accuracy, and recovery data for the proposed method.

Diltiazem concentration (*µ*g/mL)	Precision (RSD, %)		Accuracy (*n* = 3) mean (RSD, %)	Recovery (*n* = 3)			
	Intraday (*n* = 5)	Interday (*n* = 5)		Target concentration (*µ*g/mL)	Calculated concentration *µ*g/mL (mean)	%	±SD
0.50	0.96	2.40	96.67 (0.91)	12.00	11.91	99.25	0.13
5.00	1.34	1.53	100.01 (1.32)	14.00	14.08	100.57	0.07
10.00	0.78	1.18	99.06 (0.67)	18.00	18.30	101.66	0.29
50.00	1.46	1.19	98.78 (0.59)				

**Table 3:** Partial and expanded uncertainties associated to the analytical results (expressed as % relative standard deviation).

Uncertainties	Diltiazem
*U*_standard_ (%)	0.29
*U*_calibration_ (%)	2.58
*U*_precision_ (%)	1.01
*U*_accuracy_ (%)	0.39
*U*_expanded_ (%)	5.63
*U*_expanded_ (*µ*g/mL)	0.52
Concentration (*µ*g/mL)	9.30

## References

[1] Altomare DF, Binda GA, Canuti S, Landolfi V, Trompetto M, Villani RD (2011). The management of patients with primary chronic anal fissure: a position paper. *Techniques in Coloproctology*.

[2] Nelson RL, Thomas K, Morgan J, Jones A (2012). Non surgical therapy for anal fissure. *Cochrane Database of Systematic Reviews*.

[3] Herzig DO, Lu KC (2010). Anal fissure. *Surgical Clinics of North America*.

[4] Medhi B, Rao RS, Prakash A, Prakash O, Kaman L, Pandhi P (2008). Recent advances in the pharmacotherapy of chronic anal fissure: an update. *Asian Journal of Surgery*.

[5] Martin J (2011). *British National Formulary*.

[6] McCalley DV (2010). The challenges of the analysis of basic compounds by high performance liquid chromatography: some possible approaches for improved separations. *Journal of Chromatography A*.

[7] Buur JL, Baynes RE, Yeatts JL, Davidson G, DeFrancesco TC (2005). Analysis of diltiazem in Lipoderm transdermal gel using reversed-phase high-performance liquid chromatography applied to homogenization and stability studies. *Journal of Pharmaceutical and Biomedical Analysis*.

[8] Sultana N, Arayne MS, Shafi N, Siddiqui FA, Hussain A (2011). Development and validation of new assay method for the simultaneous analysis of diltiazem, metformin, pioglitazone and rosiglitazone by RP-HPLC and its applications in pharmaceuticals and human serum. *Journal of Chromatographic Science*.

[9] Sultana N, Arayne MS, Shafi N (2007). A validated method for the analysis of diltiazem in raw materials and pharmaceutical formulations by rp-HPLC. *Pakistan Journal of Pharmaceutical Sciences*.

[10] Li K, Zhang X, Zhao F (2003). HPLC determination of diltiazem in human plasma and its application to pharmacokinetics in humans. *Biomedical Chromatography*.

[11] Quaglia MG, Donati E, Fanali S, Bossù E, Montinaro A, Buiarelli F (2005). Analysis of diltiazem and its related substances by HPLC and HPLC/MS. *Journal of Pharmaceutical and Biomedical Analysis*.

[12] Georgita C, Albu F, David V, Medvedovici A (2008). Nonlinear calibrations on the assay of dilitiazem and two of its metabolites from plasma samples by means of liquid chromatography and ESI/MS^2^ detection: application to a bioequivalence study. *Biomedical Chromatography*.

[13] Zendelovska D, Stafilov T, Stefova M (2003). High-performance liquid chromatographic determination of diltiazem in human plasma after solid-phase and liquid-liquid extraction. *Analytical and Bioanalytical Chemistry*.

[15] Sheldon RA (2012). Fundamentals of green chemistry: efficiency in reaction design. *Chemical Society Reviews*.

[16] Afshar M, Salkhordeh N, Rajabi M (2013). An ecofriendly and stability-indicating HPLC method for determination of permethrin isomers: application to pharmaceutical analysis. *Journal of Chemistry*.

[17] Sun X, Jin Z, Yang L (2013). Ultrasonic-assisted extraction of procyanidins using ionic liquid solution from *Larix gmelinii* bark. *Journal of Chemistry*.

[18] Naeemullah, Kazi TG, Shah F (2012). A green preconcentration method for determination of cobalt and lead in fresh surface and waste water samples prior to flame atomic absorption spectrometry. *Journal of Analytical Methods in Chemistry*.

[19] Stocka J, Tankiewicz M, Biziuk M, Namieśnik J (2011). Green aspects of techniques for the determination of currently used pesticides in environmental samples. *International Journal of Molecular Sciences*.

[20] Tobiszewski M, Mechlińska A, Namieśnik J (2010). Green analytical chemistry—theory and practice. *Chemical Society Reviews*.

[21] dos Santos Pereira A, David F, Vanhoenacker G, Sandra P (2009). The acetonitrile shortage: is reversed HILIC with water an alternative for the analysis of highly polar ionizable solutes?. *Journal of Separation Science*.

[22] Tobiszewski M, Namieśnik J (2012). Direct chromatographic methods in the context of green analytical chemistry. *TrAC-Trends in Analytical Chemistry*.

[23] Brettschneider F, Jankowski V, Günthner T (2010). Replacement of acetonitrile by ethanol as solvent in reversed phase chromatography of biomolecules. *Journal of Chromatography B*.

[24] Desai AM, Andreae M, Mullen DG, Banaszak Holl MM, Baker JR (2011). Acetonitrile shortage: use of isopropanol as an alternative elution system for ultra/high performance liquid chromatography. *Analytical Methods*.

[25] Forster LI (2009). Conclusions on measurement uncertainty in microbiology. *Journal of AOAC International*.

[26] Konieczka P, Namieśnik J (2010). Estimating uncertainty in analytical procedures based on chromatographic techniques. *Journal of Chromatography A*.

[28] Hanson KL, VandenBrink BM, Babu KN, Allen KE, Nelson WL, Kunze KL (2010). Sequential metabolism of secondary alkyl amines to metabolic-intermediate complexes: opposing roles for the secondary hydroxylamine and primary amine metabolites of desipramine, (S)-fluoxetine, and N-desmethyldiltiazem. *Drug Metabolism and Disposition*.

[29] Tache F, Albu M (2007). Specificity of an analytical HPLC assay method of metformin hydrochloride. *Revue Roumaine de Chimie*.

[30] Quintela M, Báguena J, Gotor G, Blanco MJ, Broto F (2012). Estimation of the uncertainty associated with the results based on the validation of chromatographic analysis procedures: application to the determination of chlorides by high performance liquid chromatography and of fatty acids by high resolution gas chromatography. *Journal of Chromatography A*.

[31] de Melo Abreu S, Correia M, Herbert P, Santos L, Alves A (2005). Screening of grapes and wine for azoxystrobin, kresoxim-methyl and trifloxystrobin fungicides by HPLC with diode array detection. *Food Additives and Contaminants*.

[32] International Organization for standardization (ISO) (1995). *Guide to the Expression on Uncertainty in Measurement (GUM)*.

[33] Ribeiro RLV, Bottoli CBG, Collins KE, Collins CH (2004). Reevaluation of ethanol as organic modifier for use in HPLC-RP mobile phases. *Journal of the Brazilian Chemical Society*.

[34] Alsante KM, Ando A, Brown R (2007). The role of degradant profiling in active pharmaceutical ingredients and drug products. *Advanced Drug Delivery Reviews*.

[35] Muszalska I, Jamszoł L, Grześkowiak D (2003). Kinetics of hydrolysis of diltiazem hydrochloride in aqueous solutions. *Acta Poloniae Pharmaceutica*.

[36] Andrisano V, Hrelia P, Gotti R, Leoni A, Cavrini V (2001). Photostability and phototoxicity studies on diltiazem. *Journal of Pharmaceutical and Biomedical Analysis*.

[37] Ermer J, Ploss H-J (2005). Validation in pharmaceutical analysis—part II: central importance of precision to establish acceptance criteria and for verifying and improving the quality of analytical data. *Journal of Pharmaceutical and Biomedical Analysis*.

